# Tackling Drug-Resistant Tuberculosis: New Challenges from the Old Pathogen *Mycobacterium tuberculosis*

**DOI:** 10.3390/microorganisms11092277

**Published:** 2023-09-10

**Authors:** Giuseppe Mancuso, Angelina Midiri, Silvia De Gaetano, Elena Ponzo, Carmelo Biondo

**Affiliations:** Mycobacteriology Unit, Department of Human Pathology, University of Messina, 98125 Messina, Italy; mancusog@unime.it (G.M.); amidiri@unime.it (A.M.); sdegaetano6@gmail.com (S.D.G.); elena.ponzo@studenti.unime.it (E.P.)

**Keywords:** M.tb pathogenesis, MDR-TB, XDR-TB, drug tolerance, new approaches

## Abstract

Antibiotics have played a crucial role in the reduction in the incidence of TB globally as evidenced by the fact that before the mid-20th century, the mortality rate within five years of the onset of the disease was 50%. The use of antibiotics has eliminated TB as a devastating disease, but the challenge of resistance to anti-TB drugs, which had already been described at the time of the introduction of streptomycin, has become a major global issue in disease management. Mismanagement of multidrug-resistant tuberculosis (MDR-TB) cases, resulting from intermittent drug use, prescription errors, and non-compliance of patients, has been identified as a critical risk factor for the development of extensively drug-resistant tuberculosis (XDR-TB). Antimicrobial resistance (AMR) in TB is a multi-factorial, complex problem of microbes evolving to escape antibiotics, the gradual decline in antibiotic development, and different economic and social conditions. In this review, we summarize recent advances in our understanding of how *Mycobacterium tuberculosis* evolves drug resistance. We also highlight the importance of developing shorter regimens that rapidly reach bacteria in diverse host environments, eradicating all mycobacterial populations and preventing the evolution of drug resistance. Lastly, we also emphasize that the current burden of this ancient disease is driven by a combination of complex interactions between mycobacterial and host factors, and that only a holistic approach that effectively addresses all the critical issues associated with drug resistance will limit the further spread of drug-resistant strains throughout the community.

## 1. Introduction

**Figure 1:**
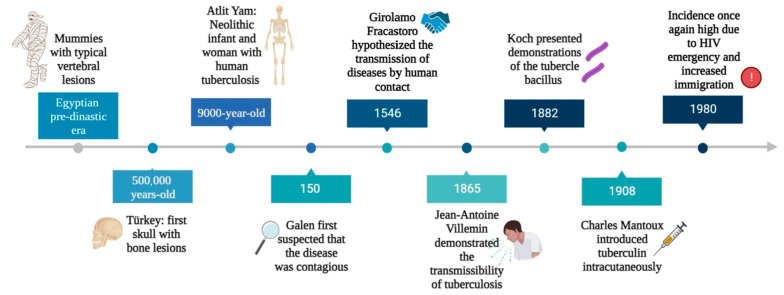
Key milestones in the history of tuberculosis.

Tuberculosis (TB) is an infectious disease caused by a group of closely related acid-fast aerobic, non-motile bacilli that belong to the *Mycobacterium tuberculosis* complex (M.TBC) [1]. M.TBC bacteria are currently grouped into nine human-adapted phylogenetic lineages (L1–L9) and four animal-associated lineages [1,2]. Of these, the L2, L3, and L4 strains are the most common worldwide [1]. M.TBC is a group of genetically similar bacteria that cause tuberculosis in a variety of hosts and comprises *Mycobacterium tuberculosis*, *Mycobacterium africanum*, *Mycobacterium bovis*, *Mycobacterium canettii*, *Mycobacterium microti*, *Mycobacterium pinnipedii*, and *Mycobacterium caprae* [3,4]. This group also includes two other species, *Mycobacterium orygis* and *Mycobacterium mungi*, that were previously designated as separate species and have been identified as the causative pathogens for TB among banded mongooses and East African oryx [5,6]. Although all these microorganisms are genetically similar, their phenotypes, host tropisms, and pathogenicity vary [4]. *M. tuberculosis* is responsible for more than 90% of human tuberculosis cases and it also is able to infect animals that come into contact with infected people [7]. Of the members of the M.TBC family, *M. bovis* has the broadest spectrum of host infection, affecting humans as well as a wide range of domestic and wild animals [8,9]. Closely related to *M. tuberculosis*, *M. canettii* and *M. africanum* rarely cause human tuberculosis in Africa and very rarely outside Africa [3,10]. The other members of M.TBC are animal-adapted strains that affect different mammalian species. These include *Mycobacterium microti* (voles), *Mycobacterium caprae* (goats and sheep), and *Mycobacterium pinnipedii* (seals and sea lions) [4,11]. It is estimated that around a quarter of the world’s population has been infected with members of *Mycobacterium tuberculosis* complex, in particular *M. tuberculosis* and *M. africanum* [12,13]. Despite significant progress in reducing the incidence and mortality rate of tuberculosis worldwide, it remains one of the leading causes of death due to infectious disease [14,15]. Tuberculosis is the oldest highly contagious disease known to mankind (Figure 1) [16]. The earliest paleopathological evidence of TB has been traced back to around 8000–10,000 years ago, corresponding to the Neolithic period, in a geographical area close to the coast of Israel [17,18]. The presence of tuberculosis in the remaining skeletons of the mother and offspring, which were buried simultaneously, was revealed by morphological analyses based on the presence of lytic lesions in the vertebral bodies [17]. However, as only 1–5% of patients with pulmonary TB develop skeletal lesions, the detection of TB by paleopathology may be greatly underestimated [19]. After this period, there is much evidence of the spread of TB in different areas of Europe, dating back to 5000 BC (Figure 1) [20]. Outside Europe, the earliest written records of tuberculosis come from China and India, dating back 2300 and 3300 years, respectively [16,21]. Tuberculosis has been present in humans since ancient times, albeit under different names: “schachepheth” in the Old Testament and “phthisis” as described by Hippocrates [22,23]. But while Hippocrates believed that tuberculosis was hereditary, Galen (131–201) was the first to suggest that it was contagious, and then Geronimo Fracastoro (1483–1553) hypothesized that infectious diseases were caused by tiny particles transmitted by human contact (Figure 1) [16,24]. In 1720, the British physician Benjamin Marten hypothesized the infectious origin of tuberculosis in his paper “A New Theory of Consumption” [16,25]. The disease was named tuberculosis by JL Schonlein in 1839 [26]. In 1865, Jean-Antoine Villemin, a French physician, demonstrated that tuberculosis was an infectious disease, but his findings were ignored by the scientific community (Figure 1) [27]. On 24 March 1882, microbiologist Robert Koch announced the discovery of *Mycobacterium tuberculosis*, the bacterium responsible for tuberculosis, at the Physiological Institute in Berlin. [26]. In 1905, he was awarded the Nobel Prize in medicine for his discovery of the bacterium that causes TB [28]. In 1890, Kock announced the discovery of tuberculin, which was the basis for Mantoux’s tuberculin skin test in 1907 (Figure 1) [28,29]. TB peaked with the onset of the Industrial Revolution, a time of increasing population density, with people living in crowded conditions, often without running water, ventilation, or sanitation [30]. During the 20th century, there was a gradual decline in the incidence of tuberculosis, particularly in developed countries [31]. This was due to several factors, the most important of which were improved living conditions, the introduction of the BCG vaccine, and the use of effective antibiotics such as streptomycin and isoniazid [32]. Until the early 1980s, the incidence of tuberculosis in the developed world was very low, but in the mid-1980s a surprising trend began to emerge: the incidence of tuberculosis was on the rise (Figure 1) [33]. Previous studies have shown that several factors have contributed to the resurgence of TB, including the emergence of the HIV epidemic, increased immigration from TB-endemic countries, and the development of multidrug-resistant (MDR) TB [34,35,36,37,38]. It is both amazing and disheartening to realize that the ancient plague, tuberculosis, found in Egyptian mummies dating back to 3000 BC, is still the world’s second deadliest infectious disease (behind COVID-19 but above AIDS) [39]. A literature review was undertaken from January 2018 until August 2023, using the following search terms: *M. tuberculosis*, Pathogenesis, MDR-TB and New Drugs on the PubMed databases. Only articles written in English and published in peer-reviewed journals were reviewed. The specific objectives of this review are to describe the mechanisms of drug resistance in *M. tuberculosis* and the ability of the bacterium to survive in a hostile environment as major factors associated with disease resurgence. Finally, new therapeutic approaches will be discussed.

## 2. Epidemiology of Tuberculosis

Although tuberculosis (TB) has been around for thousands of years, it is still one of the world’s biggest killers [1]. For centuries, tuberculosis has been a major killer of people living in poverty, overcrowding, and malnutrition, but in recent years the threat posed by this infectious disease has faded from public awareness [40]. To understand the reasons for the reduced focus on this infectious disease, it is important to look at the global trend in TB incidence over recent decades. By the mid-20th century, TB incidence and mortality had fallen in developed countries as a result of a better understanding of the disease and improvements in living conditions [41]. In addition, since the 1940s, the discovery of effective anti-tuberculosis drugs, which can significantly reduce the mortality rate due to this infection, has greatly accelerated this belief [42]. However, drug-resistant strains of TB began to emerge in the 1980s, leading the World Health Organization (WHO) to declare the problem a global health emergency in 1993 [43]. In addition, as a result of impaired cell-mediated immunity in HIV-infected patients, the incidence of TB began to rise again after decades of decline in the high-burden countries, such as areas of Sub-Saharan Africa [44]. Between 1995 and 2013, the detection rate of TB cases increased from 46% to 64% [45]. Since 1997, the WHO has published an annual Global Tuberculosis (TB) Report, which assesses the global TB situation and summarizes progress in the prevention, diagnosis, and treatment of the disease at all levels (national, regional and global) [46]. South-East Asia (43%), Africa (25%), and the Western Pacific (18%) have the highest proportion of TB cases. Smaller percentages occur in the Americas and Europe [15]. A major challenge in reducing tuberculosis mortality rates in developing countries is the difficulty in accessing health services, obtaining a diagnosis, and adhering to treatment regimes. Furthermore, diagnosis in these countries is often based on relatively insensitive methods, which are unable to detect drug-resistant strains. HIV has been the most important factor in the resurgence of tuberculosis (TB), particularly in Africa, and TB is a leading cause of death among people living with HIV worldwide (at least one-third of HIV-infected people also have TB) [47]. 

As part of its efforts to control TB, the WHO adopted the “Strategy to End TB” in 2014, with the aim of ending TB as a global public health threat by 2035 [48]. The End TB Strategy has had a significant impact, as shown by official data indicating that TB incidence and mortality have decreased by at least 20% and 35%, respectively, compared to the rates in 2015. [46]. There has also been an estimate of 49 million lives saved by 2015 [49]. However, despite the progress made, the decline in incidence has been disappointing and TB remains a major cause of morbidity and mortality in many countries [50]. The high number of TB deaths can be attributed to several factors: (1) one in three people with TB is not known to the health system and is therefore not treated; (2) treatment success rates remain too low in high-burden countries; and (3) rates of multidrug-resistant tuberculosis (MDR-TB), defined as resistance to the two main TB drugs, isoniazid and rifampicin, are increasing worldwide, with the emergence of TB caused by strains resistant to all current drugs [50,51]. The pillars of the WHO Strategy to End TB include three main lines of action to interrupt the trajectory of the TB epidemic: (1) integrated, patient-centered care and prevention; (2) bold policies and support systems; (3) intensification of research and innovation [46]. Although significant progress has been made in reducing the burden of TB, the rate of reduction remains slow and projections suggest that the global burden of TB as a global public health threat may not be eliminated by 2035, as envisaged in the Strategy to End TB [50]. According to the WHO, tuberculosis is the leading cause of death from infectious diseases worldwide, with about ten million new cases and 1.8 million deaths each year [49,52]. In 2021, there has been a decline in the diagnosis of TB and access to TB treatment as a result of the well-known COVID-19 pandemic [53,54]. The WHO estimates that 10.6 million people worldwide contracted TB in 2021, an increase of 4.5 per cent compared to 2020, causing 1.6 million deaths (including 187,000 HIV-positive people) and reversing years of global progress in reducing deaths from the disease [55]. MDR-TB is one of the leading causes of death due to antimicrobial resistance, accounting for one in three antimicrobial resistance-related deaths [56]. The number of MDR-TB infections is particularly worrying in China, India, and the Russian Federation [56,57].

## 3. Insights into Pathogenesis of Tuberculosis

**Figure 2:**
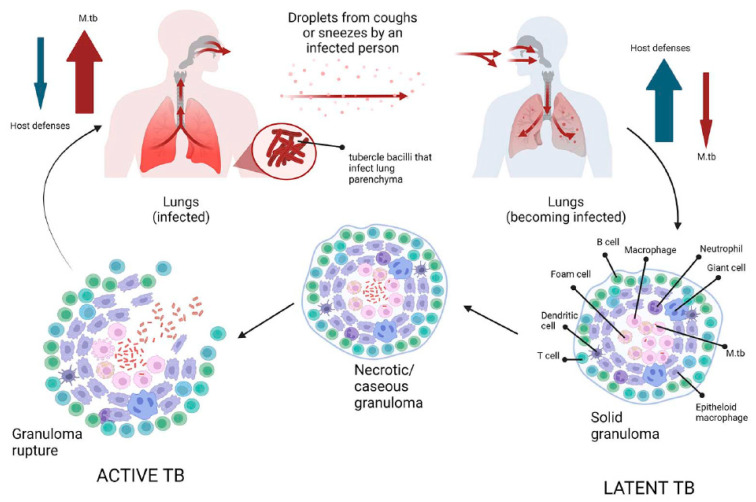
Schematic representation of tuberculosis pathogenesis. Infection with *Mycobacterium tuberculosis* occurs when aerosol droplets containing tubercle bacilli from an actively infected person reach the alveoli of an uninfected contact. In immunocompetent individuals, the host immune system fights infection by continuously recruiting and accumulating different morphotypes of macrophages, dendritic cells, B and T cells to form solid granulomas. This structure contains the bacilli, making healthy individuals latently infected (latent tuberculosis). As the disease advances, the granulomas undergo necrotic lysis of the immune cells, resulting in changes, and the center of the granuloma becomes caseous, which can lead to cavities in the lungs in the later stages of TB. If, for some reason, the host’s immune system fails to control the infection (such as in HIV or diabetes), the tubercle bacilli become active again, multiply, break out of the granuloma, and spread to other people, initiating the symptomatic phase of the disease (active TB).

Tuberculosis is an infectious bacterial disease caused by *Mycobacterium tuberculosis* (M.tb), which is airborne and most commonly affects the lungs (known as pulmonary TB), but can also spread to other parts of the body (known as extrapulmonary TB) [34]. As M.tb is essentially only found in humans (there is no animal reservoir for it), it has evolved to persist in humans for long time and only a fraction of people infected will develop active tuberculosis (about one quarter of the world’s population is latently infected with M.tb according to the WHO 2022 report) [52,58]. After initial infection, about 90% of infected people do not develop active disease, and M.tb can persist in the body for years (even a lifetime) without causing disease [58]. People with latent TB infection have no symptoms, are not contagious, and cannot spread TB to others. However, without treatment, the dormant mycobacteria can wake up and develop TB disease (active TB) in about 5% to 10% of infected people at some point in their lives [59]. The estimated lifetime risk of TB reactivation is much higher in immunocompromised patients, particularly those co-infected with HIV [60]. Tuberculosis is spread from person to person by aerosol droplets containing *M. tuberculosis* that are expelled from infected people when they cough, sneeze, or talk [61]. These tiny particles (≤5 microns in diameter), known as droplet nuclei, can remain suspended in the air for several hours in some conditions and can be transported more than 1 m. Inhaled infectious droplets travel through the respiratory tract and reach the alveoli of the lungs, where the tubercle bacilli are taken up by alveolar macrophages (AMs) of the host’s innate immune system [62]. Whether infection results in bacterial eradication, containment, asymptomatic infection, or active disease depends on the initial interaction between bacilli and AMs [62]. Thus, not all people exposed to an infectious TB patient will become infected with *M. tuberculosis*. The likelihood of TB transmission depends on several factors, the most important of which are: (1) the inhaled dose of infectious particles, which in turn depends on the bacillary load in the sputum of the patient with active TB; (2) the environment in which the exposure occurred (e.g., unventilated rooms increase the risk of droplet transmission); (3) the proximity of the individual to an infectious TB patient; (4) the duration of exposure (people in close contact with TB patients increase the risk of droplet transmission) [62,63,64]. If the macrophages fail to kill the bacilli, infected AMs migrate from the alveolar space into the lung interstitium, where the bacilli infect other cells such as DCs and different macrophage populations (Figure 2) [64]. The spread of bacilli from the site of infection is based on their ability to convert these antimicrobial cells into a permissive cellular niche [65]. At this stage, the bacteria can spread to any part of the body (e.g., lymph nodes, lungs, spine, bones, or kidneys) via the lymphatic and hematogenous pathways [66]. Numerous previous studies have shown that despite their host-protective role, AMs serve as a niche not only for M.tb growth, but also for facilitating the translocation of bacilli from the alveolar space into the interstitium prior to the arrival of recruited myeloid cells [67,68,69]. These studies clearly suggest that in the M.tb-infected lung, at least two macrophage subtypes are recruited to the site of infection and that M.tb has evolved several mechanisms that allow it to exploit the heterogeneity and plasticity of macrophages for productive infection and spread [69]. The M1 (pro-inflammatory)/M2 (anti-inflammatory) polarization of macrophages plays a crucial role in how TB infection progresses or regresses as a result of the responses they exert [69]. Moreover, recent studies have shown that at least four different subsets of macrophages which do not exhibit typical characteristics of either the M1 or M2 sublineages are involved as M.tb-permissive and M.tb-restrictive macrophage subsets [67]. Some macrophages can control infection more effectively than other cells by using anti-microbial mechanisms including phagolysosomal fusion, autophagy, and oxidative stress [65]. M.tb survives in macrophages by inhibiting phagosome maturation and phagolysosome fusion. In addition, two different forms of macrophage cell death have been described following M.tb infection: necrosis (a form of death that results in cell lysis) and apoptosis (a form of cell death that leaves the cell membrane intact) [70]. While apoptosis of infected macrophages allows bacterial replication to be controlled and is subsequently associated with reduced pathogen viability, necrosis represents a mechanism that allows bacteria to evade host defenses and spread [65,71]. Infected macrophages secrete chemokines and cytokines that activate neutrophils, which in turn release reactive oxygen species (ROS) and neutrophil extracellular traps (NETs) to kill *M. tuberculosis* [72]. To establish infection, *M. tuberculosis* inhibits ROS production by neutrophils which act as a niche for *M. tuberculosis* replication [72]. At the same time, the bacilli activate a cascade of immune responses, recruiting DCs that phagocytize and transport M.tb to the draining lymph nodes to activate the T-cell-mediated immune response [73]. Acquired cell-mediated immunity develops within 2–10 weeks of infection by stopping the multiplication of bacteria and preventing their further spread [74]. Immune cells, first infected macrophages, and neutrophils, then T and B lymphocytes, sequester M.tb in a granulomatous structure [67]. Bacterial control is established and the inflammatory response is reduced, leading to latent TB infection (LTBI) [75]. During this phase (known as primary infection), specific immunity develops, a positive skin reaction to tuberculin or an interferon-gamma release test is observed, but there are no clinical signs of TB, no culturable bacilli, and no manifestations of the disease [75]. Patients are infected with M.tb but do not have TB disease. In summary, primary infection can have several outcomes: (1) it can be eliminated by the host immune system; (2) it can progress to active disease, manifesting in less than 10% of infected individuals within 1–2 years of infection (more common in individuals co-infected with HIV or in the presence of other risk factors such as diabetes, obesity, and alcoholism); (3) it can be contained as a latent infection due to the ability of mycobacteria to enter a non-replicating persistent state in which they are resistant to therapy [76]. An effective adaptive immune response is required for the formation of granulomas, which are the result of the initial aggregation of macrophages (Figure 2) [77]. There are three main types of granulomas, representing different stages of a continuum: solid, M.tb-containing granulomas; necrotic granulomas, typical of early stages of active TB; and caseous granulomas, in late stages of TB [76]. The solid granuloma is composed of different macrophage morphotypes (epithelioid macrophages, foamy macrophages, multinucleated giant cells) and DCs, which form the central scaffold around which other cell populations, such as B and T lymphocytes, are arranged in concentric layers. Solid granulomas predominate in LTBI; these structures prevent the pathogen from spreading throughout the organism, but also allow M.tb to survive for decades by remaining in a slowly replicating state [78]. As the disease progresses, the granuloma undergoes a series of morphological changes due to the differentiation of macrophages first into epithelioid cells and then into multinucleated giant cells. The core, which consists of cell debris resulting from the necrotic lysis of host immune cells, becomes increasingly necrotic, often hypoxic, and forms a cheese-like structure known as a caseous granuloma (Figure 2) [78]. In the late stages of the disease, macrophages can transform into foam cells, either as a result of lipid droplet accumulation caused by dysregulation of host lipid metabolism or the deposition of mycolic acids [79]. Mature granulomas are dense aggregates of macrophages surrounded by an outer sheath of infiltrating lymphocytes, dominated by T and B cells. In summary, in the presence of an effective adaptive immune response, granulomas control and even sterilize infection by sclerosing and calcifying (solid granuloma) [64]. Conversely, a weak immune response results in the formation of a caseous granuloma, which acts as a reservoir, storing and harboring tubercle bacilli that, when the caseous core softens, cavitates and releases the bacilli, spreading not only to other organs but also to other people. This initiates the symptomatic phase of the disease, leading to active TB (Figure 2) [80]. Several virulence genes of *Mycobacterium tuberculosis*, which are crucial for pathogen survival and play a crucial role in its pathogenicity, have been identified [4]. Based on their function, these virulence factors can be divided into two primary groups. The first group includes virulence genes that encode enzymes involved in lipid pathways and proteins of signal transduction systems. The second category comprises virulence genes that facilitate the survival of mycobacteria in the hostile environment of host macrophages. An in-depth description of the mycobacterial virulence determinants has been reported in previous extensive reviews [4,81,82].

## 4. Effects of SARS-CoV-2 Infection on Latent TB

The risk of developing active TB disease is 18 times higher in people co-infected with HIV and TB [47]. Similarly, TB infection increases HIV replication and can accelerate the progression of HIV infection to AIDS [47]. While the mechanisms by which HIV reactivates latent TB are known, the impact of Severe Acute Respiratory Syndrome Coronavirus 2 (SARS-CoV) infection on latent TB is less clear [54]. SARS-CoV is a novel coronavirus that emerged in Wuhan, China, in December 2019 and caused the pandemic respiratory disease known as coronavirus disease 19 (COVID-19) [83]. COVID-19 was the fifth leading cause of death worldwide, and in the three years since the start of the pandemic, COVID-19 has killed more than 7.3 million people worldwide [84]. In 2020/2021, as a result of the COVID-19 health crisis, a significant number of patients with active TB were not treated and/or diagnosed, leading to a significant increase in TB mortality compared with the previous two-year period [84]. Recent clinical studies indicate that SARS-CoV-2 infection may reactivate latent TB, however, earlier data from infected M.tb mice suggest that prior M.tb infection provides protection against SARS-CoV-2-induced disease [85,86]. A recent multinational study involving 175 centers in 34 countries reported a mortality rate of 11% in patients with concomitant TB/COVID-19, twice the maximum mortality estimated for patients with TB alone [87]. While the identity and levels of cytokines produced upon infection with SARS-CoV-2 or M.tb have been identified, there are limited data on hyperinflammation, immune dysregulation, and extensive lung damage after co-infection with SARS-CoV-2 and M.tb [88]. Further studies are needed to determine how treatment against COVID-19 affects TB progression and vice versa.

## 5. Treatment of LTBI and TB Disease

**Figure 3:**
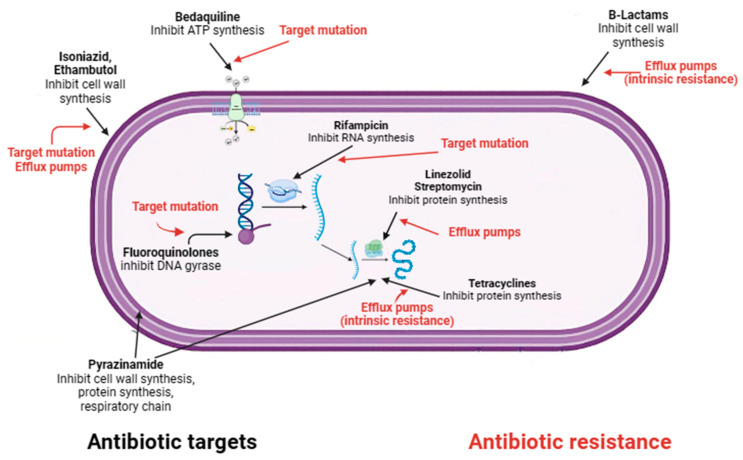
Mechanisms of action and resistance of antibiotics in *M. tuberculosis*.

Although many people can be infected with TB, not all will develop the disease. Depending on whether the host’s immune system is able to fight the bacteria and prevent its growth, there are two TB-related conditions: latent TB infection and TB disease [62]. If the immune system fails to stop the TB bacteria from growing, they will start to multiply in the body and cause TB disease [28]. Because not all people exposed to *Mycobacterium tuberculosis* develop latent tuberculosis, and even people with latent tuberculosis have no symptoms and are not infectious, there are two main ways of diagnosing LTBI: the historical tuberculin skin test and interferon gamma release assay test [58]. Neither of these tests is able to distinguish between an active and a latent TB infection [58]. International guidelines recommend testing for latent TB only in people at high risk of TB infection, such as those who work in hospitals or have other medical conditions (e.g., insulin-dependent diabetes) or a weakened immune system (e.g., HIV infection) [44]. The current recommended treatment regimen for latent TB is shorter than that for active TB and includes a short course of rifamycin therapy of three to four months (previously, it was nine months of isoniazid monotherapy) [89]. For people who cannot take a rifamycin-based regimen because of drug intolerance, the CDC recommended treatments include: (a) six months (for HIV-negative adults and children) or nine months of daily isoniazid; (b) three months of once-weekly isoniazid plus rifapentine [90]. Short-course regimens have both higher completion rates and a lower risk of hepatotoxicity than prolonged isoniazid monotherapy [91]. It is important to note that if treatment is not taken regularly or stopped too early, the bacteria can grow again and become resistant to the drugs. Unlike latent TB, the currently recommended treatment for any form of active TB (as mentioned above, TB can affect other parts of the body besides the lungs, including the lymph nodes, various organs, bones and joints, and even the brain) is the administration of long-term antibiotic therapy [91]. The most common regimens for active TB disease, according to recent WHO recommendations, involve the administration of more than one antibiotic over a period of four to nine months to ensure that all slow-growing TB bacteria are killed [92]. TB drugs fall into two categories: drugs for drug-susceptible TB (DS-TB), also known as ‘first-line TB drugs’ and drugs for drug-resistant TB (DR-TB), also called ‘second-line TB drugs’ [93]. The most common regimen for active DS-TB is isoniazid (INH) in combination with three other drugs: rifampicin (RIF), pyrazinamide (PZA), and ethambutol (EMB) (Figure 3). It is possible to recover from active TB, but patients need to take medication for many months even after symptoms disappear (at least 6 months under direct observation treatment (DOT) [93]. The disease is often fatal if left untreated (about 50 per cent of HIV-uninfected people and almost all HIV-positive people die of TB without proper treatment) [47]. The four front-line anti-TB drugs target M.tb via different mechanisms of action [78]. Briefly, INH is a prodrug that, after activation by katG, a mycobacterial catalase/peroxidase enzyme, inhibits the synthesis of mycolic acids which are essential components of the mycobacterial cell wall [94]. Rifampicin, also known as rifampin, exerts its antimicrobial activity by forming a stable complex with the bacterial DNA-dependent RNA polymerase, thereby inhibiting RNA synthesis [95]. The mechanism of action of PZA is only partially understood, despite its clinical use as an anti-tuberculosis drug for 50 years. Pyrazinamide (PZA) is a drug that is converted to pyrazinoic acid (POA) by the bacterial enzyme pyrazinamidase. POA inhibits the bacterial synthesis of coenzyme A, an essential cofactor in metabolism. PZA exerts its effect by penetrating into all types of TB lung lesions, including necrotic caseous granulomas, and by killing the non-growing bacilli [96]. Ethambutol is a bacteriostatic agent that inhibits the arabinosyl transferases (embA, embB, and embC), which are essential for the synthesis of the bacterial cell wall arabinogalactan and lipoarabinomannan [97]. An in-depth description of the mechanisms of action of four drugs that target M.tb mechanisms of action has been reported elsewhere [78]. Although treatment with all “first-line TB drugs” for 2 months, followed by 4 months of isoniazid and rifampicin, which is used to treat DS-TB, had an overall success rate of 86% in 2019, there are still unresolved problems with the duration of this therapy [78,98]. In fact, despite the gradual reduction in the duration of therapy required to treat DS-TB from 24 to 6 months, non-compliance or dropout remain major barriers to effective treatment. Unfortunately, this depends on the various adverse effects associated with this treatment, including hepatitis, skin reactions, gastrointestinal intolerance, neurological, and hematological toxicity [78].

## 6. The Complexity of Drug Resistance in M.tb

Despite the availability of the WHO-short-course regimen that cured 85% of patients in 6 months, 1.6 million deaths were attributed to TB in 2021 [99]. About half a million of the 10.6 million people with TB in 2021 had multidrug-resistant tuberculosis (MDR-TB), defined as infection caused by bacilli resistant to at least isoniazid and rifampicin, the two effective first-line drugs against TB [100]. The emergence of drug-resistant strains around the world poses a major threat and could jeopardize the End TB Strategy’s efforts to achieve absolute reductions of 95% in TB mortality and 90% in TB new cases by 2035 [15]. Although drug resistance is widely recognized as a major challenge to effective TB control worldwide, the underlying causes of drug-resistant TB (e.g., poor adherence to treatment, prescription of the wrong regimen, and re-infection by M.tb, are among the most important) have yet to be addressed [101]. In this regard, of the 132,222 documented MDR-TB cases worldwide in 2020, only 33% received appropriate treatment and only 59% were completed [101]. In 2021, the WHO revised the drug classification for MDR-TB and introduced new definitions for pre-extensively drug-resistant tuberculosis (pre-XDR) and extensively drug-resistant tuberculosis (XDR-TB) [102]. The updated classifications help determine the optimal treatment for patients in relation to the severity of their TB. Managing drug-resistant tuberculosis (DR-TB) can be complicated because the approach to treatment and prognosis depends heavily on the resistance pattern. Pre-XDR-TB is an intermediate stage between MDR-TB and XDR-TB, i.e., MDR-TB that is also resistant to a fluoroquinolone or a second-line injectable agent [102]. XDR-TB is a rare type of MDR TB that exhibits additional resistance to at least one fluoroquinolone and any of the injectable second-line TB drugs [102]. The definitions of pre-XDR and XDR-TB indicate a worsening severity of the disease caused by resistance to multiple drugs. Similar to other bacteria, *Mycobacterium tuberculosis* develops antibiotic resistance through several intrinsic and acquired mechanisms [103]. M.tb exhibits intrinsic resistance to a broad spectrum of antibiotics, which is attributed to its thick, waxy, and hydrophobic cell envelope [104]. The lipid-rich nature of the cell wall makes it extremely hydrophobic, and the low number of porins prevents the penetration of hydrophilic compounds [104]. In addition, the distinctive structure of the mycobacterial cell wall hinders the movement of hydrophobic compounds, including the macrolide, rifamycin, tetracycline, and fluoroquinolone. This implies that factors other than lipophilicity also influence the transport of molecules across the mycobacterial cell wall [104]. Again, with regard to intrinsic factors, mutations in genes encoding drug targets or enzymes that activate them are the main mode of drug resistance and occur mainly through single nucleotide polymorphisms and insertion-deletions [105,106]. Unlike other bacterial pathogens, acquisition of drug resistance by horizontal gene transfer has not been reported in *M. tuberculosis*. Most clinically relevant drug resistance in *M. tuberculosis* is conferred by chromosomal mutations rather than by resistance plasmids, in addition to the intrinsic resistance mechanisms discussed above [104]. Depending on the antimicrobial, several resistance mechanisms may exist, the most common of which are: drug target alteration, abrogation of prodrug activation, overexpression of drug targets, and overexpression of efflux pumps (Figure 3) [107]. For example, isoniazid resistance is associated with mutations in several genes including *katG*, *inhA*, *ahpC*, *kasA*, and *NDH*, although the two main molecular mechanisms of resistance involve gene mutations in *katG* and *inhA* or its promoter region [108]. In addition to resistance caused by target mutations, other mechanisms have been described that allow the pathogen to acquire drug resistance despite apparent genetic susceptibility [109]. This phenotypic drug resistance is associated with the formation of a heterogeneous population of bacteria characterized by differences in a state of slow growth, DNA replication, metabolism, and efflux of compounds [110]. This heterogeneous population, known as persisters, can become phenotypically tolerant to antimycobacterial drugs without acquiring genetic mutations [105]. In addition, recent studies have shown that the propensity of mycobacterial populations to contain subpopulations of cells in different physiological states at different stages of infection may be related to the limited efficacy of current TB treatments [111]. This may influence the emergence of persisters and create an environment that favors the development of drug-tolerant populations [112]. The evolution of drug-resistant M.tb is also thought to be driven by epistasis, a genetic event in which the phenotypic effect of a mutation changes depending on whether other mutations exist in the same genome [105]. Depending on the effect on bacterial fitness, this genetic event may have positive or negative consequences. Positive epistasis promotes the evolution of MDR by minimizing its cost (e.g., interacting mutations have a lower fitness cost than genetic determinants alone), whereas negative epistasis restricts the evolution of MDR by increasing its cost [113]. A large number of studies have shown that positive epistasis is a very common phenomenon in mycobacteria [105,114]. It is important to note that MDR isolates in which positive epistasis confers fitness advantages are associated with increased transmissibility and are thus frequently encountered in the clinical setting, while those in which epistatic interactions result in decreased fitness are associated with low transmission rates and are rarely encountered in the clinical setting [114].

## 7. New Insights into the Treatment of MDR-TB 

Drug-resistant TB poses a significant global threat to TB control [45]. According to the WHO’s latest global TB report, there were an estimated 450,000 cases of DR-TB in 2021, an increase of 3.1% from 437,000 in 2020 [99]. New anti-tuberculosis drugs are needed to provide more treatment options. Natural products, particularly marine metabolites, have received considerable attention in drug discovery research over the past few decades. Among the natural products and their derivatives known to have antimycobacterial activity, fucoxanthin and lichen-derived psoromic acid (PA) have shown the greatest potential as drugs to combat tuberculosis infections [115,116]. These two natural products show significant inactivation of two crucial enzymes, UGM and TBNAT, involved in *Mycobacterium tuberculosis* cell wall biosynthesis [115,116]. The management of MDR-TB requires medication regimes consisting of second-line drugs, including bedaquiline and fluoroquinolones (Figure 3) [117]. These regimes are longer, more expensive, and induce more frequent side-effects than first-line treatments for drug-susceptible tuberculosis. Management of pre-XDR-TB and XDR-TB is considered even more challenging [93]. In recent years, the availability of new drugs with innovative mechanisms of action has made it possible for the first time to use shorter, all-oral MDR-TB regimens, similar to the traditional 6-month regimen used for DS-TB [78]. In December 2002, WHO published an updated version of the guidelines for the treatment of drug-resistant TB, which includes two new recommendations: (1) Instead of the 9-month regimen for MDR-TB patients, a 6-month regimen of bedaquiline, pretomanid, linezolid, and moxifloxacin (BPaLM) is recommended (Figure 3). This treatment includes extensive pulmonary TB and extrapulmonary TB, but excludes TB involving the central nervous system, miliary TB, and osteoarticular TB; (2) For patients with MDR-TB who are not eligible for treatment with BPaLM and who are not resistant to fluoroquinolones, a 9-month all-oral regimen is recommended rather than a longer regimen (18 months) [92,117]. In all cases, where shorter regimens cannot be used due to adverse drug reactions, extensively drug-resistant TB, drug–drug interactions, severe forms of extrapulmonary TB, past relapses, and longer regimens (18 months) remain a viable option [117]. The latter remain largely unchanged from previous guidelines and involve the use of drugs classified into three groups (A, B, and C) based on the certainty of the evidence on the efficacy and safety of treatment [92]. Although these new guidelines recommending shorter oral regimens for MDR-TB represent a revolution in the treatment of MDR-TB, a number of issues need to be addressed to allow full implementation of the new recommendations: (a) the high cost of bedaquiline, pretomanid, and linezolid limits access to these drugs for many patients; (b) the short regimens include only four drugs, which carry a high risk of treatment failure in the event of resistance to one or more of these drugs; (c) universal access to drug susceptibility testing to rule out fluoroquinolone resistance needs to be implemented to allow appropriate regimen selection and to avoid potentially toxic drugs in the event of resistance [118].

## 8. Discussion

Tuberculosis is a chronic infectious disease for which there was no effective cure until the first half of the 20th century. Deeper comprehension of the disease and the discovery of effective antibiotics for tuberculosis have accelerated patients’ recovery and reduced the prevalence and transmission of tuberculosis cases. The emergence of drug-resistant strains of tuberculosis in the late 1980s prompted the WHO to announce the disease as a global health emergency in 1993. Mismanagement of drugs used to treat MDR-TB, including prescription errors and poor patient compliance, led to the emergence of XDR-TB in 2006. With nearly 10 million incident cases and 1.5 million deaths worldwide in 2020, tuberculosis (TB) remains the leading cause of death among humans due to a single pathogen. As an additional challenge for TB control, in 2020, nearly half a million cases of rifampicin (RMP)-resistant (RR) TB were reported, out of which 78% were multidrug resistant (at least INH- and RMP-resistant). In 2021, the WHO revised the definition of XDR-TB as tuberculosis resulting from strains with MDR-TB, which are resistant to fluoroquinolones and at least one other second-line injectable drug used to treat tuberculosis (levofloxacin, moxifloxacin, bedaquiline, or linezolid). As a result, the treatment of XDR-TB is less effective and the mortality rate of infected patients is higher than that of MDR-TB. The recognition that *M. tuberculosis* has developed resistance to all drugs used in clinical practice has catalyzed research into the development of new drugs and regimens in recent years (currently, numerous anti-TB drugs have received approval for clinical trials). It is also essential to understand how the bacterium gains drug resistance or evades chemotherapy, despite its established genetic susceptibility. Furthermore, the persistence of *M. tuberculosis* within macrophages and the response of various cell populations to *M. tuberculosis* infection are well-documented, with some myeloid populations limiting the growth of the bacterium and others promoting or not inhibiting its spread. However, the molecular mechanisms responsible for these responses remain unclear. A thorough understanding of these processes at a molecular level is essential to enable the development of new host-targeted therapies and vaccines. To achieve global TB control, attention should be given to the following: (1) accurate use of antibiotics during TB treatment; (2) appropriate prophylaxis for the contacts of drug-resistant cases; (3) developing novel and precise rapid diagnostics; (4) creating shorter regimens that efficiently eliminate all mycobacterial populations, including persistent microorganisms; and (5) discovering new approaches to TB drug development. Only a comprehensive approach that addresses all aspects of drug resistance will have a positive impact on the eradication of the disease.

## Data Availability

Not applicable.
